# 4^th^ Stage Transvaginal omental herniation during VBAC complicated by shoulder dystocia: a unique presentation of uterine rupture

**DOI:** 10.1186/1471-2393-13-76

**Published:** 2013-03-23

**Authors:** Aruyaru Stanley Mwenda

**Affiliations:** 1 Transmara District Hospital, P.O Box 98–40700, Kilgoris, Kenya

## Abstract

**Background:**

Uterine rupture is a common complication in women attempting their first virginal birth after caesarean section (VBAC) but the risk diminishes with subsequent VBACs. It occurs in rates of 0.5-9% and is influenced by various factors.

**Case presentation:**

A unique case of uterine rupture in a Kenyan woman of African descent during a repeat VBAC complicated by shoulder dystocia was discovered during the 4^th^ stage of labour when omentum was noted protruding through the vagina. She had delivered 4 years earlier by caesarean section.

**Conclusion:**

It is not common to experience uterine rupture among women attempting repeat VBAC. When it occurs, it may not always follow the known pattern intra-partum and is often associated with poor foetal outcome.

## Background

Uterine rupture, defined as full thickness break in the wall of the uterus [1] is the commonest and most serious complication of vaginal birth after cesarean delivery (VBAC) [2]. It occurs in rates of 0.5-9% [2,3] and is influenced by type of scar, number of scars, induction of labour, increasing maternal age, foetal macrosomia and interdelivery interval less than 18–24 months [2-4]. It is common in women attempting their first VBAC after primary caesarean section (CS) but the risk diminishes with subsequent VBACs [4,5].

There are few reports of ruptured uterus after a repeat VBAC [2] available in English literature. In this article, a unique case of uterine rupture during a repeat VBAC complicated by shoulder dystocia was discovered during the 4^th^ stage of labour when omentum was noted protruding through the vagina.

## Case presentation

**Figure 1:**
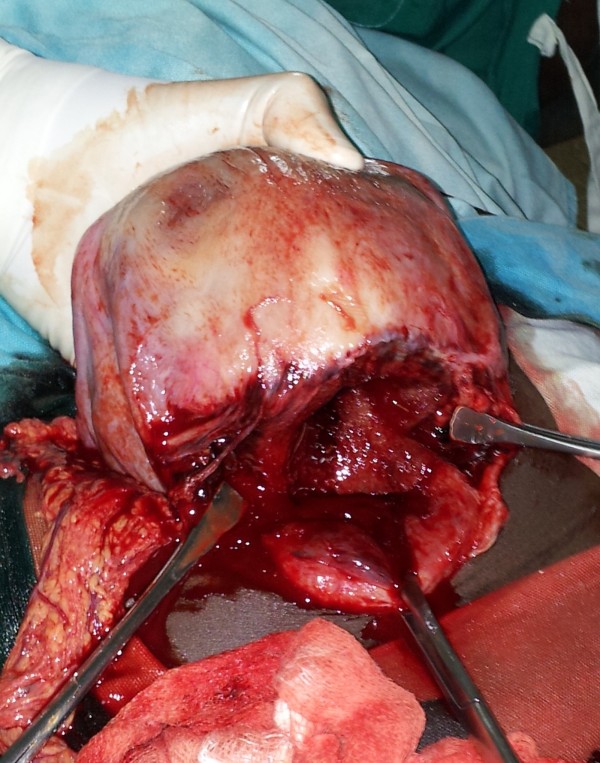
Transverse tear along the previous scar.

A Kenyan lady of African descent presented in second stage of labour. She had had a caesarean delivery 4 years earlier due to prolonged labour and an uneventful vaginal birth two years later. On examination she was mildly pale, had a blood pressure of 117/70mmHg and a pulse of 90 beats per minute. Haemogram indicated a haemoglobin of 10.2 g/dl and platelets of 270 000 per microliter. She was having strong uterine contractions and the presenting part had fully descended. No foetal heart rate was discernible on auscultation. There was no augmentation of labour or fundal pressure. The midwives conducted labour but there was shoulder dystocia. The author was called and did a posterior arm delivery after failed McRobert’s manoeuvre. A 3200gm lifeless infant was delivered. A diagnosis of uterine rupture was made when the omentum was noted prolapsing into the vagina after delivery of the placenta. The patient was prepared for laparotomy under spinal anaesthesia. She had normal oxygen saturations and blood pressure intra-operatively. Half a litre of haemoperitoneum was encountered. A 10 cm full thickness tear along the lower segment transverse scar (Figure 1) was repaired in layers. The operation was uneventful. Based on clinical assessment by the anaesthetist and the doctor, she was started on a unit of whole blood post operatively which progressed to completion without any transfusion reaction. Eight hours post operatively, and 2 hours after completion of transfusion, she suddenly developed shortness of breath, tachycardia and sweating and died within 30 minutes while undergoing resuscitation. The exact cause of the mortality could not be ascertained and the relatives opted not to have a post mortem done.

## Conclusion

While a common complication during the first VBAC, uterine rupture decreases during the subsequent VBACs [5]. When present, ruptured uterus is often associated with induction/augmentation of labour [2-4,6,7], fundal pressure [6] and some form of labour dystocia especially after 7 cm of cervical dilatation [1,5]. Intrapartum, the point of uterine rupture is usually indicated by onset of foetal bradycardia and tender maternal abdomen [4,6].

Shoulder dystocia has been reported in association with uterine rupture, both as a precipitant [7] and as a result [6]. There are also recorded cases of transvaginal omental and bowel herniation after third stage as the first signs of uterine rupture [7,8].The case by Sighal and colleagues [7] was associated with prostaglandin E2 labour induction and fundal pressure while Guasch and colleagues [8] reported a case of previous repeated uterine curettage as the possible risk for the uterine rupture.

This patient could not tell the exact indication for the caesarean section she had undergone, only saying she had laboured for a day at a health centre before she was referred to a hospital for the caesarean section. Vacuum delivery was not attempted prior to the operation. Her first and second babies weighed 3500 grams and 3000 grams respectively. Since both prior deliveries did not occur in our hospital, it was not possible to cross check her medical records. Neither was it possible to establish if a partograph was used during her first delivery and what pattern of labour it showed. The decision to transfuse the patient despite a near-normal haemoglobin and mild pallor may have been unnecessary for this patient.

The uniqueness of this case lies in its presentation in the form of vaginal omental herniation. That the woman presented with no perceivable foetal heart rate means the rupture could have occurred earlier whence foetal life was lost. The bradycardia and subsequent loss of foetal heart rate occurs due to placental detachment and uterine contraction [4] following rupture. If this had already happened to this patient, it is logical to imagine that the foetus would have been expelled through the 10 cm tear into the peritoneal cavity. There is also the possibility that the shoulder dystocia could have led to the uterine rupture. If this was the case, then the cause of foetal demise remains unknown. The placenta was not yet detached and active management of third stage of labour was done. Overzealous vaginal delivery in the setting of shoulder dystocia can also cause uterine rupture [6].

With excellent haemodynamic status intra-op, the sudden post-operative death of the mother is a shock in an operation gone just well. The last recorded blood pressure was 100/82mmHg, less than an hour before death. The shortness of breath and tachycardia, coupled with the rapidity of deterioration of cardiorespiratory status in the hands of resuscitating staff points to a possibility of pulmonary embolism. Pulmonary embolism is a leading cause of mortality following gynaecologic surgery and cesarean section [9]. It is also likely to have been due to reaction to the blood transfusion.

In this part of Kenya, it is not uncommon to find women presenting to hospital in advanced labour, not to mention those delivering at home. Had this patient presented earlier for delivery at hospital, probably a non-reassuring foetal heart rate would have been picked and the baby saved through an emergency caesarean delivery. As there was no post mortem examination, the exact cause of death could not be established.

It is not common to experience uterine rupture among women attempting repeat VBAC. Uterine rupture is often associated with poor foetal outcome. When it occurs, it may not always follow the known pattern intra-partum. It is important that women with a previous caesarean delivery present to hospital for delivery so that good labour monitoring and appropriate intervention is done. This was a unique case of a successful second VBAC complicated with uterine rupture that did not present classically.

## Consent

Written informed consent was obtained from the patient’s husband for publication of this case report and any accompanying images. A copy of the written consent is available for review by the Editor of this journal.

## Competing interests

The authors declare that they have no competing interests.

## Pre-publication history

The pre-publication history for this paper can be accessed here:

http://www.biomedcentral.com/1471-2393/13/76/prepub
